# Exploration into the Syntheses of Gallium‐ and Indiumborates under Extreme Conditions: *M*_5_B_12_O_25_(OH): Structure, Luminescence, and Surprising Photocatalytic Properties

**DOI:** 10.1002/anie.201804083

**Published:** 2018-07-26

**Authors:** Daniela Vitzthum, Klaus Wurst, Johann M. Pann, Peter Brüggeller, Markus Seibald, Hubert Huppertz

**Affiliations:** ^1^ Institut für Allgemeine, Anorganische und Theoretische Chemie Leopold-Franzens-Universität Innsbruck Innrain 80–82 6020 Innsbruck Austria; ^2^ OSRAM Opto Semiconductors GmbH Mittelstetter Weg 2 86830 Schwabmünchen Germany

**Keywords:** borates, high-pressure synthesis, luminescence, photocatalytic hydrogen production, structure elucidation

## Abstract

Explorative solid‐state chemistry led to the discovery of the two new compounds Ga_5_B_12_O_25_(OH) and In_5_B_12_O_25_(OH). Extreme synthetic conditions within the range of 12 GPa and a temperature of 1450 °C realized in a Walker‐type multianvil apparatus resulted in the formation of an unprecedented tetragonal structure with the exclusive presence of condensed BO_4_ tetrahedra, forming cuboctahedral cavities. Doping of these cavities with Eu^3+^ in In_5_B_12_O_25_(OH) yielded in an orange–red luminescence. Photocatalytic tests of In_5_B_12_O_25_(OH) revealed a hydrogen production rate comparable to TiO_2_ but completely co‐catalyst free.

The transition from limited fossil fuels to renewable energy sources is one of the main challenges of humankind. In our timescale, the sun presents an eternal and plentiful source of energy. Ways of direct solar to chemical energy conversion have been investigated since the first findings of Fujishima and Honda in 1972.^1^ Although hydrogen is a conveniently usable fuel, for example, in hydrogen fuel cells, the storage of the volatile gas is an unresolved issue. A possible solution may be the storage in form of a hydrocarbon, for example, methanol. Hydrogen is then retrieved from the storage molecule through a reformation process. To enable this hydrogen liberation, metal borates gained significance in the recent years as possible photocatalysts, a result amongst other things of their great stability. Especially triel borates not only shifted into our research focus, but have been examined for their photocatalytic activity lately by other groups as well.^2^ Unlike most other compounds, our newly discovered indium borate In_5_B_12_O_25_(OH) produces hydrogen quite effectively without a co‐catalyst as explained herein.

Apart from its interesting properties, In_5_B_12_O_25_(OH) is a member of the new borate structure type *M*_5_B_12_O_25_(OH), which is (besides *M*BO_3_)^3^ the second known borate structure incorporating both gallium and indium as metal cations. In the system of In‐B‐O‐H borates, three other compounds (H_2_InB_5_O_10_, In_19_B_34_O_74_(OH)_11_, and InB_6_O_9_(OH)_3_)^4^ have been published in the years 2010, 2016, and 2018 indicating the actuality of this research field. For the system Ga‐B‐O‐H, Ga_5_B_12_O_25_(OH) is now besides Ga_9_B_18_O_33_(OH)_15_⋅H_3_B_3_O_6_⋅H_3_BO_3_ 5 and Ga_2_B_3_O_7_(OH)^6^ the third known hydroxylated borate.

Herein we elucidate the discovery of the two new compounds *M*_5_B_12_O_25_(OH) (*M*=Ga, In) synthesized via high‐pressure/high‐temperature syntheses, their crystal structures, Raman and IR spectroscopic investigations, and the surprising results of the photocatalytic experiments with In_5_B_12_O_25_(OH). Additionally, energy dispersive X‐ray spectroscopy (EDX) and luminescence measurements of the europium‐doped indium compound are presented.

*M*_5_B_12_O_25_(OH) (*M*=Ga, In) crystallizes in the tetragonal space group *I*4_1_/*acd* (no. 142, origin choice 2) with eight formula units (*Z*=8) per unit cell. The lattice parameters of the quite large unit cells of both isotypes are shown in Table S2 (see Supporting Information). Interestingly. these borates share great structural similarities with a high‐pressure oxonitridophosphate synthesized by Marchuk et al. in 2014.^7^ A brief comparison of the compounds is given in the Supporting Information.

In *M*_5_B_12_O_25_(OH) (*M*=Ga, In), the metal cations constitute two crystallographically different octahedra and in accordance with these harsh high‐pressure conditions all boron atoms are coordinated by four oxygen atoms each, composing large corner‐sharing networks. For the visualization of the metal–oxygen polyhedra and their correlation with the hydrogen bonds, Ga_5_B_12_O_25_(OH) was chosen, because for this compound the proton could be located via the difference Fourier syntheses. Figure 1 shows the arrangement of the GaO_6_ octahedra in and throughout the unit cell. Both, the green and orange polyhedra represent the isolated double units of edge‐sharing Ga1O_6_ octahedra. Along the crystallographic *c* axis, every second unit is displaced along *b* or rotated by 90° pertaining to the corresponding double‐entity. The distorted Ga2O_6_ octahedra with the half‐occupied, deflected Ga2 positions in the center are positioned along the $\bar{4}$ inversion axis (light‐blue in Figure 1). Each Ga2O_6_ octahedron is surrounded by four Ga1_2_O_10_ double‐entities and connected to one of these through a hydrogen bond. There are four possible hydrogen atom sites as depicted in Figure 2, but since the hydrogen atom has an occupancy of a quarter, only one of these positions is occupied at a time. It seems likely that when, referring to Figure 2, one of the lower hydrogen bonds is formed, the upper Ga2 atom is occupied and vice versa. Although the hydrogen atom in In_5_B_12_O_25_(OH) could not be located during the single‐crystal structure refinement, a similar situation is expected, as the In2‐octahedron shows the same distortion as Ga2. The *M*−O bond lengths in the octahedra of Ga2 and In2 are hence slightly longer as in the regular Ga1 and In1 octahedra, respectively. With average interatomic distances of 1.97 and 2.15 Å and individual values ranging from 1.909(2)– 2.065(2) Å for Ga1 and 2.069(2)–2.247(2) Å for In1, the distances are in good agreement with those reported in the literature.3b, 4b, 6, 8 The distorted octahedra exhibit with 2.10 and 2.36 Å larger average distances and also quite uncommon maximal lengths of 2.307(2) Å for Ga2 and 2.748(2) Å for In2. All bond lengths and angles for the metal octahedra as well as for the hydrogen bonds can be found in the Tables S5–S7 in the Supporting Information. The network of corner‐sharing BO_4_ tetrahedra in this new borate structure type is rather complicated as there are 96 BO_4_ tetrahedra built up of three crystallographically different boron atoms in the unit cell. Alternately, a set of twelve corner‐sharing BO_4_ tetrahedra forms either a cuboctahedral cage or two six‐membered curved strings that enlace the distorted In2O_6_ and accordingly Ga2O_6_ octahedra. The cuboctahedral cages can be looked upon as a tetrahedral arrangement of four dreier rings^9^ which in doing so additionally form four sechser rings. Both of these described structural motifs and their alternative arrangement throughout the unit cell are visualized in Figure 3. Illustrations showing the displacement ellipsoids of all atoms are given in the Supporting Information (Figures S2 and S3). All BO_4_ tetrahedra show reasonable bond lengths and angles as can be checked in the Tables S5 and S6 in the Supporting Information. The cuboctahedral cages are with a diameter of approximately 5.4 Å large enough to accommodate a rare earth cation like Eu^3+^, Sm^3+^, or even Ce^3+^. In Ga_5_B_12_O_25_(OH), these cavities are empty, whereas in the Eu^3+^‐doped Indium analogue, electron density indicating an integration of 2 % Eu could be found. Hence, Eu^3+^ was positioned in the center of these cuboctahedral cages with a site occupancy factor of 0.02, which means every 50th cavity in In_5_B_12_O_25_(OH):Eu^3+^ is statistically occupied with europium. The refinement of such a small amount of activator ion is remarkable and was only possible because Eu^3+^ is not competing with In^3+^ but fills otherwise empty cavities in the structure.

**Figure 1:**
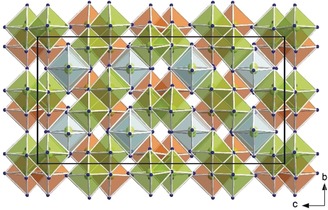
Visualization of the GaO_6_ octahedra in Ga_5_B_12_O_25_(OH). The green and orange polyhedra center Ga1 forming isolated, edge‐sharing double‐entities. The light blue polyhedra surround the half‐occupied, dislocated Ga2 positions.

**Figure 2:**
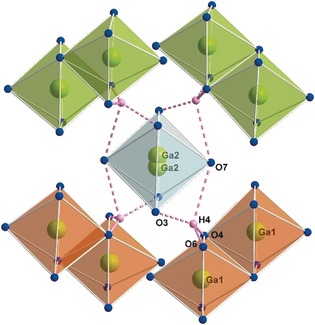
The hydrogen bonds in Ga_5_B_12_O_25_(OH) are formed by O4 as donor atom and O3, O7, and O6 as acceptors. Only one of the four hydrogen bonds and one of the two Ga2 atoms shown in the picture are present at a time. The Ga1 atoms form isolated double‐entities consisting of two edge‐sharing octahedra each. The colors of the octahedra match the color code in Figure 1.

**Figure 3:**
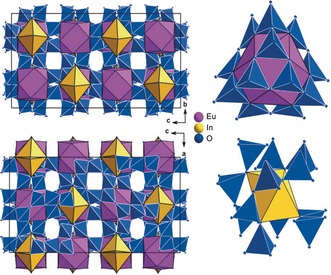
Visualization of the BO_4_ tetrahedra network in the unit cell of In_5_B_12_O_25_(OH) viewed in the directions $\left[\bar{1}00\right]$ and $\left[0\bar{1}0\right]$ . Alternately, twelve corner‐sharing BO_4_ tetrahedra form cuboctahedral cages and six‐membered curved strings surrounding Eu^3+^ and In2^3+^, respectively and thus form a kind of a three‐dimensional checkerboard pattern. The pink polyhedra display the cuboctahedral cavities of which in In_5_B_12_O_25_(OH):Eu^3+^ approximately every 50th is statistically occupied with an Eu^3+^ ion.

To our surprise, the compound In_5_B_12_O_25_(OH) showed extraordinary high performance for photocatalytic hydrogen production from methanol. The rate of hydrogen evolution was determined to be 220±20 μmol h^−1^ g^−1^ (*s*=11 μmolh^−1^ g^−1^, *N*=12, *p*=95 %). Although the sample was not phase pure, comparative experiments with the byproduct InB_6_O_9_(OH)_3_ showed no hydrogen evolution at all on the timescale of interest.4c Therefore, the photocatalytic activity stems from In_5_B_12_O_25_(OH) and by accounting for the inactive byproduct InB_6_O_9_(OH)_3_, the rate can be estimated to be even higher. Figure 4 shows the hydrogen production over 12 h of our sample as well as the background measurement of pure methanol.

**Figure 4:**
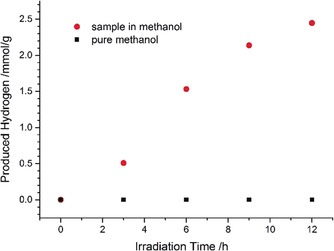
Hydrogen production: Irradiation of 0.30 mg of an In_5_B_12_O_25_(OH) sample in 5 mL methanol with a 700 W Hg medium pressure lamp.

A discussion of the mechanism has been given for a comparable borate structure.^6^ It has been clearly shown that these borates have semiconductor properties and therefore the conduction band delivers electrons. The photocatalytic conditions were adapted to UV light, since an irradiation above 300 nm does not contribute to the hydrogen production. Therefore, it can be concluded that the band gap is in the region of 4.1 eV. The semiconductor plays the combined role of light absorber and proton reduction catalyst. Methanol delivers electrons as the sacrificial donor and oxidation products thereof have been found via quadrupole mass spectrometry. This means that the band gap and the band edges are suitable for H^+^ reduction and methanol oxidation, thus no further component is necessary and the system is co‐catalyst free.

TiO_2_ as catalyst for photocatalytic hydrogen production was thoroughly studied and improved in an ongoing effort for many years. Although the comparison of literature is delicate because of different illumination setups, up to recent publications the activity of In_5_B_12_O_25_(OH) is superior or on a level with precious‐metal‐doped TiO_2_.^10^ Lin and co‐workers showed an activity of roughly 30 μmol h^−1^ g^−1^ for Pt‐loaded TiO_2_.^11^ Compared to other recently published borates of the 13th group of the periodic table, the herein described In_5_B_12_O_25_(OH) shows a high hydrogen evolution rate even without commonly employed co‐catalysts, such as Ni, Pt, or Ru.^2^, 4b, 5, 6, 12

Previously published catalysts with hydrogen evolution rates of 2.84b and 120 μmol h^−1^ g^−1^ 6 show the development of new photocatalytically active phases. The approach of explorative solid‐state chemistry under extreme conditions lets us expect new and interesting catalysts to be prepared in the near future, since there is a huge space for the catalytic fine‐tuning of this class of compounds. Potential applications are widespread, from a primary energy source via artificial photosynthesis, to hydrogen production from room‐temperature methanol reformation.

To test its luminescence properties, a powder sample containing In_5_B_12_O_25_(OH):Eu^3+^ was excited using a 460 nm laser. The resulting luminescence spectrum exhibits typical peaks for Eu^3+^ emission as shown in Figure 5. The predominant intensity between 580–620 nm confirms the orange–red luminescence impression of the powder sample. Based on literature comparisons, the common ^5^D→^7^F transitions for Eu^3+^ were assigned as following: The most intense peaks at 585 and 600 nm can be attributed to ^5^D_0_→^7^F_1_ transitions, the subsequent weaker peaks most likely stem from ^5^D_0_→^7^F_2_ transitions, and the little bump at 698 nm can be explained with ^5^D_0_→^7^F_4_ transitions.^13^ The relative intensities of the ^5^D_0_→^7^F_1_ transitions are significantly higher than those of the ^5^D_0_→^7^F_2_ transitions, which is characteristic for (pseudo)centrosymmetric Eu^3+^ positions in the crystal structure.^14^ In contrast to the sidephase InB_6_O_9_(OH)_3_ 4c in the examined powder sample, In_5_B_12_O_25_(OH):Eu^3+^ is centrosymmetric and the Eu^3+^ ion could be located at the symmetric Wyckoff position 8*a*. Therefore, and because europium could not be detected via EDX in a single‐crystal of the side phase InB_6_O_9_(OH)_3_ originating from the two‐phase powder sample, we claim that the luminescence spectrum (see Figure 5) is representative for In_5_B_12_O_25_(OH):Eu^3+^.

**Figure 5:**
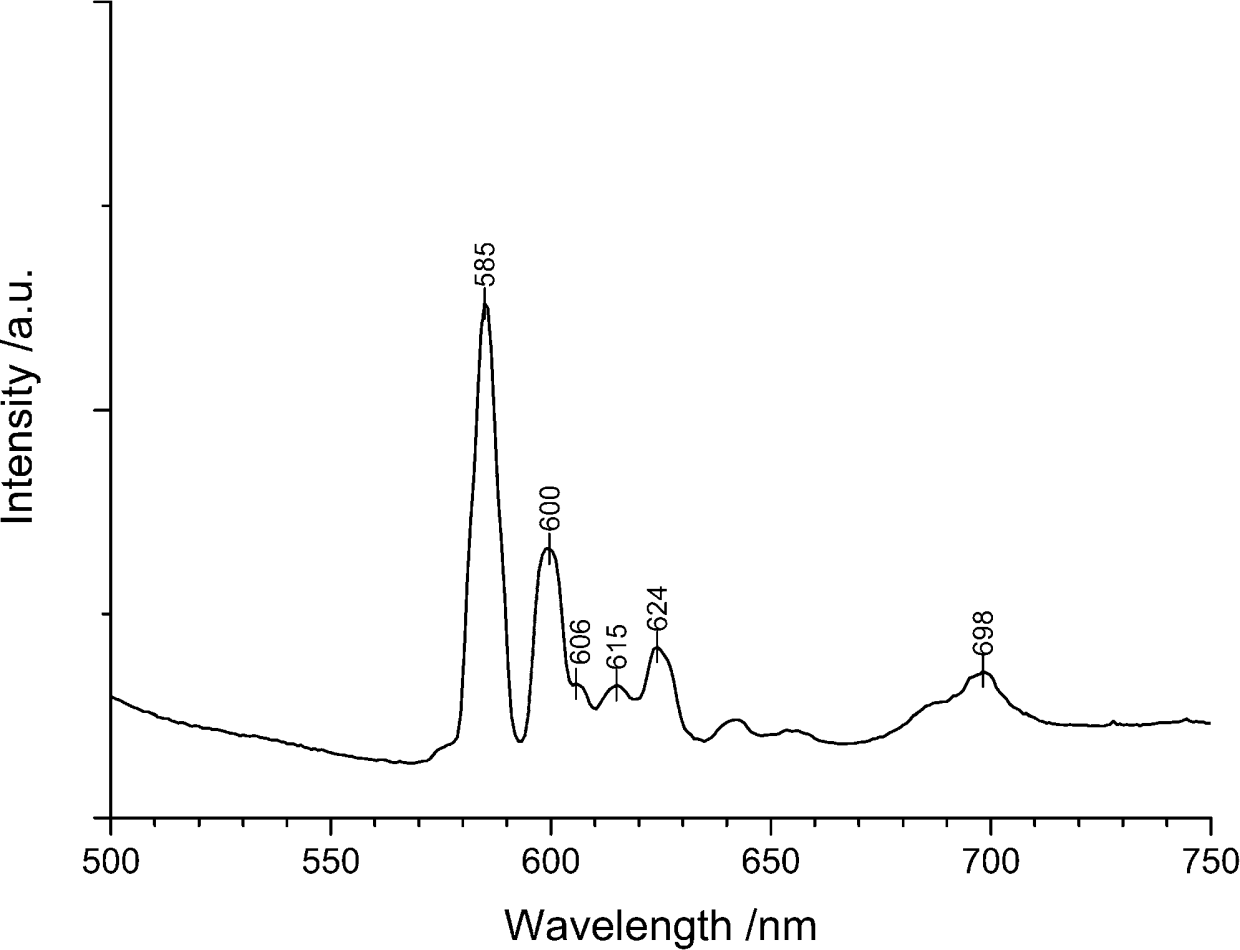
Luminescence spectrum of In_5_B_12_O_25_(OH):Eu^3+^.

To confirm the presence of europium in In_5_B_12_O_25_(OH):Eu^+3^, EDX was performed on a single‐crystal, which had in advance been tested on a single‐crystal X‐ray diffractometer to be the desired phase by determining its lattice parameters. The analyzed single‐crystal of In_5_B_12_O_25_(OH):Eu^3+^ clearly contained europium. However, the semiquantitative measurement under low vacuum did not really allow to specify quantitatively the amount of Eu. A picture of the examined single‐crystal as well as the EDX spectrum and the expected and measured elemental ratios can be found in the Supporting Information (Figure S4, S5 and Table S8).

The single‐crystal IR and Raman spectra of Ga_5_B_12_O_25_(OH) and In_5_B_12_O_25_(OH) can be found in the Supporting Information (Figures S6, S7). Typical vibrations of InO_6_ or GaO_6_ octahedra appear at the lowest wavenumbers up to about 800 cm^−1^.4b, 6, 15 While those peaks in the IR spectra overlap with bands of the BO_4_ bending and stretching vibrations, usually occurring at 800–1150 cm^−1^, they can be distinguished in the Raman spectra.^16^ In both, the Raman and IR spectra, peaks at high wavenumbers confirm the presence of the protons. According to Hammer et al.,^17^ the hydrogen bonds, which could be determined for Ga_5_B_12_O_25_(OH), lie with an average D–A distance of 2.74 Å in the crossover between weak and strong hydrogen bonds and should therefore appear around 3200 cm^−1^.

Herein, we reported on the new borate structure type *M*_5_B_12_O_25_(OH) (*M*=Ga, In), which could be synthesized under extreme high‐pressure/high‐temperature conditions with either Ga^3+^ or In^3+^ as metal cations. In its large unit cell, the structure type comprises various interesting structural motifs, such as cuboctahedral tetrahedra‐formations or isolated edge‐sharing octahedra double‐units. The indium‐containing compound could be doped with 2 % Eu^3+^ and showed luminescence when irradiated with a suitable laser. During the refinement, it was possible to locate the Eu^3+^ ions in the otherwise empty cuboctahedral vacancies built up by the BO_4_ tetrahedra cages. Furthermore, In_5_B_12_O_25_(OH) was tested for its photocatalytic activity and indeed produced significant amounts of hydrogen without a co‐catalyst without being degraded itself. In our assessment, the research field of high‐pressure indium and gallium borates has only just opened, promising not only further structure types to be discovered, but also possible applications, such as the demonstrated photocatalytic properties.

## Experimental Section

Ga_5_B_12_O_25_(OH) and its indium isotype were both synthesized via solid state reaction in a multianvil press under extreme conditions of 11.0 GPa and 1450 °C for Ga_5_B_12_O_25_(OH) and 12.2 GPa and 1450 °C for In_5_B_12_O_25_(OH). Experimental details of the syntheses can be found in the Supporting Information. Both compounds could not be obtained phase‐pure. The best synthesis of Ga_5_B_12_O_25_(OH) was carried out with β‐Ga_2_O_3_ (Strem Chemicals, Kehl, Germany, 99.9 %) and H_3_BO_3_ (Carl Roth, Karlsruhe, Germany, 99.5 %) in the stoichiometric ratio of Ga:B=1:2.4 according to Equation (1). The reaction product additionally contained GaBO_3_ 18 and an unidentified byproduct.

(1) $$5\;\mathrm{Ga}{}_{2}O{}_{3}\;+\;24\;H{}_{3}\mathrm{BO}{}_{3}\to 2\;\mathrm{Ga}{}_{5}B{}_{12}O{}_{25}\left(\mathrm{OH}\right)\;+\;35\;H{}_{2}O$$

Concerning phase purity—the most successful synthesis of In_5_B_12_O_25_(OH) was achieved by a molar ratio of In:B=1:1.8 starting from In_2_O_3_ (ChemPUR, Karlsruhe, Germany, 99.9 %) and H_3_BO_3_ encapsulated in gold foil. As side phase, InB_6_O_9_(OH)_3_ 4c formed. The phase fractions were determined via Rietveld^19^ (see Supporting Information). For the synthesis of the europium doped In_5_B_12_O_25_(OH):Eu^3+^, approximately 1.5 weight % Eu_2_O_3_ was added to the educt mixture. Detailed initial weights can also be found in the Supporting Information. All products appeared as clean‐colorless powders, the product of the Eu containing synthesis showed orange–red luminescence under UV light excitation.

## Conflict of interest

The authors declare no conflict of interest.

## Supporting information

As a service to our authors and readers, this journal provides supporting information supplied by the authors. Such materials are peer reviewed and may be re‐organized for online delivery, but are not copy‐edited or typeset. Technical support issues arising from supporting information (other than missing files) should be addressed to the authors.

Supplementary
