# Supplementary material for: Exploration into the Syntheses of Gallium‐ and Indiumborates under Extreme Conditions: M 5B12O25(OH): Structure, Luminescence, and Surprising Photocatalytic Properties
Source: Angew Chem Int Ed Engl. 2018 Jul 26;57(35):11451–5. doi: 10.1002/anie.201804083 (PMC6120471; doi:10.1002/anie.201804083)
Supplement: Supplementary file 1 — Supplementary [file ANIE-57-11451-s001.pdf]

Angewandte Chemie
Eine Zeitschrift der Gesellschaft Deutscher Chemiker

Supporting Information

Exploration into the Syntheses of Gallium- and Indiumborates under Extreme Conditions: $M_5B_{12}O_{25}(OH)$: Structure, Luminescence, and Surprising Photocatalytic Properties

*Daniela Vitzthum, Klaus Wurst, Johann M. Pann, Peter Brüggeller, Markus Seibald, and Hubert Huppertz**

anie_201804083_sm_miscellaneous_information.pdf

Supporting Information

TABLE OF CONTENTS

1 Experimental Part
1.1 Synthesis Description
1.2 X-ray Powder Diffraction Analysis
1.3 Powder Pattern
1.4 Rietveld
1.5 Crystal Structure Analysis
1.6 Photocatalytic Experiments
1.7 Luminescence Spectroscopy
1.8 Elemental Analysis
1.9 Vibrational Spectroscopy
2 Results and Discussion
2.1 Crystallographic Data
2.1.1 Crystal Data and Structure Refinement
2.1.2 Atomic Coordinates
2.1.3 Displacement Parameters
2.1.4 Bond Lengths
2.1.5 Bond Angles
2.1.6 Hydrogen Bonds
2.1.7 Illustration of Displacement Ellipsoids
2.1.8 Structural Comparison with $CaMg_2P_6O_3N_{10}$
2.2 EDX
2.2.1 BSE Image
2.2.2 EDX Spectrum
2.2.3 Atom Ratios
2.3 IR Spectra
2.4 Raman Spectra
3 References

1 Experimental Part

1.1 Synthesis Description

Setup for the performance of the high-pressure/ high-temperature syntheses

The reaction mixture was filled into a boron nitride crucible (*α*-BN, Henze Boron Nitride Products AG, Kempten, Germany). This crucible constitutes the center of a "14/8" assembly, which is further surrounded by eight beveled tungsten carbide cubes (Hawedia, Marklkofen, Germany).The compression and heating was performed with a multianvil device based on a Walker-type module (Voggenreiter, Mainleus, Germany). A detailed description of this high-pressure/ high-temperature setup can be found in the literature.[1]

Synthesis of $Ga_5B_{12}O_{25}(OH)$

0.23229 g Ga_2O_3 and 0.36691 g H_3BO_3 were ground and about 35 mg of it prepared as above described. The maximum pressure of 11 GPa was built up in 267 min, then the heating process started. The maximum temperature of 1450 °C was reached in 7 min, held for 5 min and afterwards down-regulated to 1000 °C in 70 min. After the heating was turned off, the decompression to ambient conditions began and took 790 min. The clean-white, crumbly product containing $Ga_5B_{12}O_{25}(OH)$, $GaBO_3$ and an unidentified byproduct revealed colorless crystals.

Synthesis of $In_5B_{12}O_{25}(OH)$

0.02257 g In_2O_3 and 0.01756 g H_3BO_3 were ground in an agate mortar and about 30 mg of the mixture encapsulated in gold foil (Sigma-Aldrich, USA, 0.025 mm, 99.99 %) before being placed in a boron nitride crucible. Otherwise, the synthesis setting was the same as described above. The maximum pressure of 12.2 GPa was reached in 325 min. The heating process started with heating-up to the maximum temperature of 1450 °C in 7 min. This temperature was held for 5 min before the heating was slowly down-regulated to 1150 °C in 40 min and afterwards turned-off completely. The decompression process took 1000 min. When the BN crucible was cut open, the Au capsule appeared as solid bullet, which means that it had melted during the heating process. The reaction product around the gold ball was clean-white and its single-crystals, belonging to $In_5B_{12}O_{25}(OH)$ and $InB_6O_9(OH)_3$, colorless. This reaction product was used for the photocatalytic experiments.

Synthesis of $In_5B_{12}O_{25}(OH):Eu^{3+}$

For the synthesis of $In_5B_{12}O_{25}(OH):Eu^{3+}$, 0.0380 g In_2O_3, 0.0311 g H_3BO_3 and 0.00105 g Eu_2O_3 were ground in an agate mortar and about 30 mg of the mixture was encapsulated in gold foil before being placed in the BN crucible and handled as above described. The extreme pressure of 13 GPa was accomplished in 421 min, the maximum temperature of 1150 °C was reached in 8 min and held for 6 min. Afterwards the heating was down-regulated to 850 °C in 40 min and finally turned off. The decompression process lasted more than 20 h. During this synthesis, the Au capsule did not melt completely, but softened up and appeared afterwards as compressed, concave pillar. The product was clean-white and revealed colorless single-crystals of $In_5B_{12}O_{25}(OH)$ and $InB_6O_9(OH)_3$. The single-crystal from which the crystal structure of $In_5B_{12}O_{25}(OH):Eu^{3+}$ was solved, stemmed from this synthesis.

1.2 X-ray Powder Diffraction Analysis

The X-ray powder diffraction patterns were collected on a Stoe Stadi P powder diffractometer with Ge(111)-monochromatized Mo-K$_{\alpha 1}$ radiation (λ = 0.7093 Å) used in transmission geometry. The diffractograms were both detected by a Dectris Mythen 1 K detector in the 2Θ range of 2–60° with a step width of 0.7 or 0.34 °2Θ and a measuring time of 15 or 28 s per step for the patterns containing $Ga_5B_{12}O_{25}(OH)$ or $In_5B_{12}O_{25}(OH)$, respectively. To date, both borates could not be synthesized phase-pure and the experimental powder patterns showed reflexes of the byproducts $GaBO_3$[2] and an unknown phase besides $Ga_5B_{12}O_{25}(OH)$ and $InB_6O_9(OH)_3$[3] next to $In_5B_{12}O_{25}(OH)$. In Figure S1 (Supplementary Information), the experimental powder patterns of $Ga_5B_{12}O_{25}(OH)$ and $In_5B_{12}O_{25}(OH)$ are compared with each other and with their theoretical patterns derived from single-crystal data. Reflections resulting from byproducts are marked with asterisks.

1.3 Powder Pattern

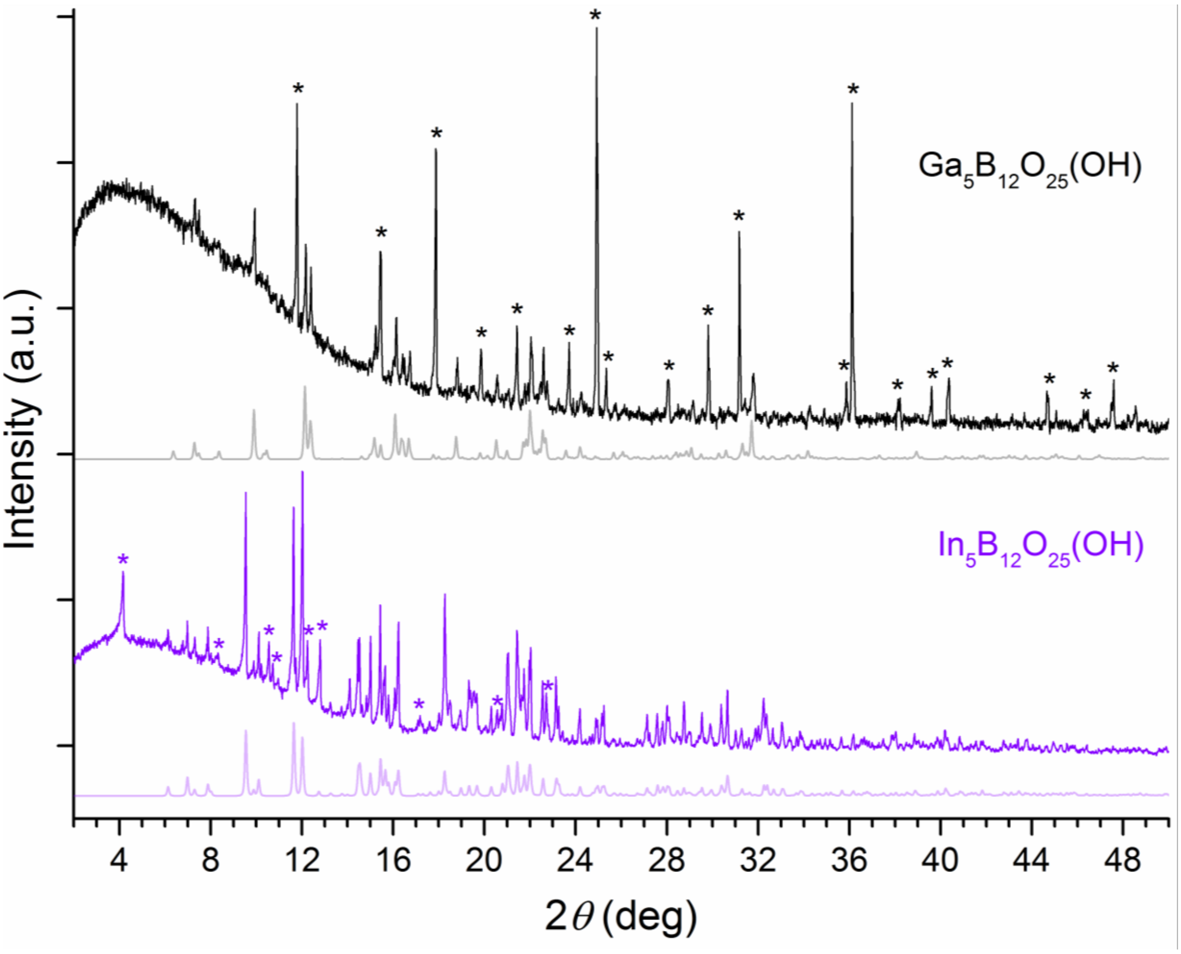

Figure S1. Experimental powder patterns of our best syntheses leading to $Ga_5B_{12}O_{25}(OH)$ (black) and $In_5B_{12}O_{25}(OH)$ (purple) compared to their theoretical patterns calculated from single-crystal data (grey and light purple), respectively. Asterisks mark the reflections of the byproducts.

1.4 Rietveld

To distinguish the amount of $M_5B_{12}O_{25}(OH)$ (M = In, Ga) in the received products, a Rietveld refinement[4] with the program TOPAS 4.2[5] was done. The synthesis product containing $Ga_5B_{12}O_{25}(OH)$ clearly showed besides $GaBO_3$ another byproduct that could not be identified. Hence, the ratio of $Ga_5B_{12}O_{25}(OH)$ could not be determined sufficiently.
$In_5B_{12}O_{25}(OH)$ was identified as the main phase with 66 % besides the byproduct $InB_6O_9(OH)_3$ with 34 %. Details of the quantitative Rietveld analysis can be found in Table S1.

Table S1. Rietveld fit for the synthesis product containing $In_5B_{12}O_{25}(OH)$.

phase 1: $In_5B_{12}O_{25}(OH)$	65.6(7) weight%
phase 2: $InB_6O_9(OH)_3$	34.4(7) weight%
R_{exp}	3.94
R_{wp}	8.71
R_p	5.83
GOF	2.21

1.5 Crystal Structure Analysis

Single-crystals of the title compounds were measured with a Bruker D8 Quest diffractometer equipped with a Photon 100 CMOS detector. For the multiscan absorption correction of the intensity data, SADABS 2014/5[6] was used, and the structure solutions and parameter refinement was performed with the SHELXS/L-2013[7] software implemented in the program WINGX-2013.3[8]. According to the systematic reflection conditions, $Ga_5B_{12}O_{25}(OH)$ and $In_5B_{12}O_{25}(OH)$ were solved and refined in the space group $I4_1/acd$ (no. 142, origin choice 2). To facilitate a comparison of the two isotypic borates, both data sets were standardized with STRUCTURE TIDY[9] implemented in PLATON[10] (version 170613). Except for the proton, whose position was located and refined with a bond restraint (d = 83 pm) being only occupied by ¼th, all atom positions in $Ga_5B_{12}O_{25}(OH)$ could be refined anisotropically. The position of the Ga2 atom has an occupancy of 0.5. In $In_5B_{12}O_{25}(OH)$, all atoms were refined anisotropically with the exception of the proton, which could not be located at all. As in the gallium containing isotype, the position In2 is only half-occupied leading to distorted $In2O_6$ octahedra. The doped Eu^{3+} position was refined with a site occupancy factor of 0.02. Details of the data collection can be found in the synoptical Table S2. The positional parameters, displacement parameters, Wyckoff positions, site occupancy factors, interatomic distances and angles are listed in the Supplementary Information in the Tables S1–5. Further details of the crystal structure investigation may be obtained from Fachinformationszentrum Karlsruhe, 76344 Eggenstein-Leopoldshafen, Germany (fax: +49-7247-808-666; e-mail: crysdata@fiz-karlsruhe.de) on quoting the deposition number CSD-434134 for $Ga_5B_{12}O_{25}(OH)$ or CSD-434135 for $In_5B_{12}O_{25}(OH):Eu_{0.02}$.

1.6 Photocatalytic Experiments

For photocatalytic activity measurements, the sample (60 mg/l) was suspended in 5 ml pure methanol (Acrös Organics, 99+ % extra pure) in a quartz glass vial and degassed via three pump thaw-cycles. The UV-irradiation was carried out with a water cooled 700 W medium pressure mercury lamp at a distance of 9 cm. The suspension was produced via sonication for 5 minutes and maintained through vigorous magnetic stirring during the irradiation process. After overnight irradiation, there was no temperature change of the sample recognizable. The hydrogen measurement was conducted with an Inficon 3000 µ-GC containing a 5 Å molsieve column, a thermal conductivity sensor, and Argon 5.0 as carrier gas. The catalytic activity was determined through the measured hydrogen gas concentration and the dilution was appropriately accounted for. Each measurement was repeated three times.

1.7 Luminescence Spectroscopy

The title compound´s emission signal was measured by exciting a powder sample with a 460 nm laser (model Sapphire 460/10, 10 mW; COHERENT). The converted light was collected using a multi-mode

optical fiber (QP 600-2-VIS/BX; Ocean Optics) and finally detected in a spectrometer (QE 65000; Ocean Optics).

1.8 Elemental Analysis

Semiquantitative EDX (energy dispersive X-ray spectroscopy) measurements were performed on a single-crystal of $In_5B_{12}O_{25}(OH):Eu^{3+}$ to prove the presence of europium. A Jeol JSM-6010LA scanning electron microscope (SEM) equipped with a Peltier-cooled Bruker XFlash 410-M silicon drift detector was used to analyze the single-crystal, which was placed on a carbon tape. As the sample was non-conducting, a low vacuum of about 30 Pa had to be used. An acceleration voltage of 15 kV was applied and the measured spot size was 44 μm^2. The output count rate came to 2000 cps. The chemical composition was measured on different spots of the single-crystal, which showed similar atom ratios.

1.9 Vibrational Spectroscopy

The transmission FT-IR spectra of $Ga_5B_{12}O_{25}(OH)$ and $In_5B_{12}O_{25}(OH)$ were measured in the spectral range of 600–4000 cm^{-1} via a Vertex 70 FT-IR spectrometer (spectral resolution 4 cm^{-1}) equipped with a KBr beam splitter, a liquid nitrogen-MCT (Mercury Cadmium Telluride) detector as well as a Hyperion 3000 microscope (Bruker, Vienna, Austria). For the measurement, the single-crystals were positioned on a BaF_2 sample holder and 120 scans of the $Ga_5B_{12}O_{25}(OH)$ single-crystal and 220 scans of the $In_5B_{12}O_{25}(OH)$ single-crystal were acquired, respectively. A Globar (silicon carbide) rod was used as a mid-IR source and a 15× IR objective as focus. OPUS 6.5 was used to correct atmospheric influences.

Raman spectroscopy was performed on single-crystals of $Ga_5B_{12}O_{25}(OH)$ and $In_5B_{12}O_{25}(OH)$ using a Labram-HR 800 Raman microscope (Horiba Jobin Yvon, Tulln, Austria) equipped with an Olympus 50× objective lens and a 1024×256 open-electrode charge-coupled device detector. The samples were excited with the 532 nm emission line of a frequency-doubled 100 mW Nd:YAG laser with a spot surface of about 1 μm in diameter and a spectral solution of about 0.6 cm^{-1}. An optical grating with 1800 lines mm^{-1} was used for the dispersion of the scattered light. The Raman spectrum of $Ga_5B_{12}O_{25}(OH)$ was measured in the range of 150–3800 cm^{-1} with an acquisition time of 50 s and background-corrected with a sixth-order polynomial function from 150–2200 cm^{-1} and manually at higher wavenumbers. The $In_5B_{12}O_{25}(OH)$ single-crystal showed different Raman intensity throughout the spectral range of 150–3800 cm^{-1}. Therefore, we collected two spectra, the first starting from 150 to 1300 cm^{-1} with an acquisition time of 50 s and the second from 1300–3800 cm^{-1} with 0.4 s, because it showed far more intensity and would otherwise have reached the detector saturation. For both spectra, the baseline was corrected with LABSPEC[11]. All measurements were performed at ambient conditions.

2 Results and Discussion

2.1 Crystallographic Data

2.1.1 Crystal Data and Structure Refinement

Table S2. Crystal data and structure refinement of $Ga_5B_{12}O_{25}(OH)$ and $In_5B_{12}O_{25}(OH)$.

empirical formula	**$Ga_5B_{12}O_{25}(OH)$**	**$In_5B_{12}O_{25}(OH)$**
Molar mass, g mol^{-1}	895.339	1123.85
Crystal system	tetragonal	
Space group	$I4_1/acd$	
Powder diffractometer	STOE Stadi P	
Radiation	Mo-$K_{\alpha 1}$ (λ = 0.7093 Å)	
Single-crystal diffractometer	Bruker D8 Quest Kappa	
Radiation	Mo-K_{α} (λ = 0.7107 Å)	
Single-crystal data:		

a, Å	11.150(5)	11.639(2)
c, Å	21.76(2)	22.282(5)
V, Å^3	2705(3)	3018(2)
Formula units per cell *Z*	8	8
Calculated density, g cm^{-3}	4.397	4.942
Crystal size, mm^3	0.050 × 0.045 × 0.035	0.040 × 0.030 × 0.030
Temperature, K	293(2)	293(2)
Detector distance, mm	50	40
Exposure time	0.5°/frame, 30 s/frame	0.5°/frame, 30 s/frame
Absorption coefficient, mm^{-1}	10.033	7.788
F(000), e$^-$	3392	4114
θ range, deg	3.18–36.00	3.08–32.49
Range in hkl	−18/+17; ±18; −33/+35	±17; ±17; ±33
Reflections total / independent	25885/1612	62655/1370
R_{int}	0.0473	0.0920
Reflections with $I \geq 2\sigma(I)$	1431	1169
R_σ	0.0211	0.0197
Data / ref. parameters	1612 / 106	1370 / 103
Absorption correction	multiscan	multiscan
Final R_1 / wR_2 [$I \geq 2\sigma(I)$]	0.0186 / 0.0430	0.0209 / 0.0382
Final R_1 / wR_2 (all data)	0.0253 / 0.0456	0.0318 / 0.0403
Goodness-of-fit on F^2	1.057	1.062
Largest diff. peak / hole, e Å^{-3}	1.47 / −0.65	2.07 / −0.67

2.1.2 Atomic Coordinates

Table S3. Wyckoff positions, atomic coordinates, isotropic U_{iso} or equivalent isotropic displacement parameters U_{eq} /Å^2 and site occupancy factors (S.O.F.) for $Ga_5B_{12}O_{25}(OH)$ and $In_5B_{12}O_{25}(OH)$. U_{eq} is defined as one third of the trace of the orthogonalized U_{ij} tensor (standard deviations in parentheses).

Atom	Wyckoff position	*x*	*y*	*z*	U_{eq} (U_{iso} for H3)	S.O.F.
**$Ga_5B_{12}O_{25}(OH)$**						
Ga1	32*g*	0.32036(2)	0.09695(2)	0.20509(2)	0.00502(5)	1
Ga2	16*f*	0.01270(4)	0.26270(4)	⅛	0.0094(2)	0.5
B1	32*g*	0.0735(2)	0.1622(2)	0.25051(6)	0.0040(2)	1
B2	32*g*	0.2540(2)	0.1824(2)	0.08195(6)	0.0040(2)	1
B3	32*g*	0.4054(2)	0.0138(2)	0.08154(6)	0.0055(2)	1
O1	32*g*	0.16120(8)	0.22924(8)	0.29014(4)	0.0044(2)	1
O2	32*g*	0.16229(8)	0.11458(8)	0.04561(4)	0.0044(2)	1
O3	32*g*	0.19235(8)	0.26479(8)	0.12568(4)	0.0048(2)	1
O4	32*g*	0.31426(8)	0.08075(8)	0.29334(4)	0.0047(2)	1
O5	32*g*	0.32661(8)	0.09573(8)	0.11742(4)	0.0042(2)	1

O6	32*g*	0.33698(8)	0.25057(8)	0.04185(4)	0.0040(2)	1
O7	16*d*	0	¼	0.03408(6)	0.0042(2)	1
H3	32*g*	0.38(2)	0.08(2)	0.31(2)	0.2(2)	0.25
**$In_5B_{12}O_{25}(OH)$**						
In1	32*g*	0.30590(2)	0.10687(2)	0.20555(2)	0.00545(5)	1
In2	16*f*	0.02119(4)	0.27119(4)	⅛	0.0146(2)	0.5
B1	32*g*	0.0928(2)	0.1852(2)	0.0000(2)	0.0040(5)	1
B2	32*g*	0.2599(2)	0.1939(2)	0.0817(2)	0.0040(5)	1
B3	32*g*	0.3976(3)	0.0230(2)	0.0802(2)	0.0056(5)	1
O1	32*g*	0.1590(2)	0.2132(2)	0.28494(8)	0.0053(3)	1
O2	32*g*	0.1633(2)	0.1360(2)	0.04881(8)	0.0045(3)	1
O3	32*g*	0.2112(2)	0.2700(2)	0.12875(8)	0.0054(3)	1
O4	32*g*	0.3095(2)	0.0786(2)	0.29811(8)	0.0052(3)	1
O5	32*g*	0.3251(2)	0.1040(2)	0.11326(8)	0.0050(3)	1
O6	32*g*	0.3385(2)	0.2553(2)	0.04111(8)	0.0049(3)	1
O7	16*d*	0	¼	0.0307(2)	0.0048(5)	1
Eu1	8*a*	½	¼	⅛	0.015(3)	0.02

2.1.3 Displacement Parameters

Table S4. Anisotropic displacement parameters U_{ij} /Å^2 of $Ga_5B_{12}O_{25}(OH)$ and $In_5B_{12}O_{25}(OH)$ (standard deviations in parentheses).

Atom	U_{11}	U_{22}	U_{33}	U_{12}	U_{13}	U_{23}
**$Ga_5B_{12}O_{25}(OH)$**						
Ga1	0.00504(7)	0.00628(7)	0.00373(6)	-0.00079(4)	0.00000(4)	0.00083(4)
Ga2	0.0120(3)	0.0120(3)	0.0043(2)	0.0050(3)	0.0000(2)	-0.0000(2)
B1	0.0038(5)	0.0045(5)	0.0036(5)	0.0007(4)	-0.0001(4)	0.0001(4)
B2	0.0047(5)	0.0036(5)	0.0037(5)	-0.0001(4)	0.0011(4)	-0.0003(4)
B3	0.0058(5)	0.0060(5)	0.0047(5)	0.0015(4)	0.0016(4)	0.0016(4)
O1	0.0050(4)	0.0035(3)	0.0047(3)	0.0001(3)	-0.0015(3)	0.0001(3)
O2	0.0040(3)	0.0042(4)	0.0048(3)	-0.0002(3)	-0.0013(3)	0.0005(3)
O3	0.0049(3)	0.0046(3)	0.0049(3)	-0.0001(3)	0.0000(3)	-0.0016(3)
O4	0.0049(4)	0.0042(4)	0.0049(3)	0.0010(3)	0.0009(3)	0.0006(3)
O5	0.0054(4)	0.0042(4)	0.0031(3)	0.0016(3)	0.0000(3)	0.0002(3)
O6	0.0045(3)	0.0032(3)	0.0044(3)	-0.0007(2)	0.0016(3)	-0.0002(3)
O7	0.0041(5)	0.0049(5)	0.0035(5)	0.0016(4)	0	0
**$In_5B_{12}O_{25}(OH)$**						
In1	0.00652(8)	0.00659(8)	0.00326(8)	0.00032(6)	-0.00002(6)	-0.00112(7)
In2	0.0208(2)	0.0208(2)	0.0022(2)	0.0002(2)	-0.0002(2)	0.0087(3)
B1	0.005(2)	0.004(2)	0.004(2)	-0.0006(9)	0.0000(9)	0.000(2)
B2	0.004(2)	0.006(2)	0.003(2)	-0.0007(9)	0.0001(9)	0.0007(9)
B3	0.005(2)	0.007(2)	0.005(2)	0.0018(9)	0.0002(9)	0.002(2)
O1	0.0062(9)	0.0046(8)	0.0051(8)	0.0005(6)	-0.0024(6)	-0.0007(6)
O2	0.0045(8)	0.0038(8)	0.0053(8)	0.0011(7)	-0.0021(6)	-0.0010(6)
O3	0.0062(8)	0.0057(8)	0.0043(8)	-0.0027(7)	0.0011(6)	-0.0015(7)
O4	0.0049(8)	0.0054(8)	0.0053(8)	0.0003(6)	0.0022(7)	0.0006(7)
O5	0.0064(8)	0.0053(8)	0.0033(8)	0.0011(7)	0.0001(6)	0.0020(7)
O6	0.0053(8)	0.0035(8)	0.0058(8)	0.0003(6)	0.0021(7)	0.0004(6)
O7	0.004(2)	0.004(2)	0.006(2)	0	0	0.0005(9)
Eu1	0.014(4)	0.014(4)	0.018(7)	0	0	0

2.1.4 Bond Lengths

Table S5. Interatomic distances /Å in $Ga_5B_{12}O_{25}(OH)$ and $In_5B_{12}O_{25}(OH)$ (standard deviations in parentheses).

		**$Ga_5B_{12}O_{25}(OH)$**	**$In_5B_{12}O_{25}(OH)$**			**$Ga_5B_{12}O_{25}(OH)$**	**$In_5B_{12}O_{25}(OH)$**
*M*1–	O5	1.909(2)	2.069(2)	*M*2–	O7	1.988(2) 2×	2.130(3) 2×
	O4	1.930(2)	2.089(2)		O3	2.003(2) 2×	2.214(2) 2×
	O1	1.952(2)	2.145(2)		O3	2.307(2) 2×	2.748(2) 2×
	O4	1.983(2)	2.160(2)	**Ø*M*2–O**		**2.10**	**2.36**
	O6	2.001(2)	2.209(2)				
	O2	2.065(2)	2.247(2)				
**Ø*M*1–O**		**1.97**	**2.15**				
B1–	O2	1.475(2)	1.464(3)	B2–	O5	1.478(2)	1.471(3)
	O6	1.481(2)	1.478(3)		O6	1.482(2)	1.472(3)
	O7	1.482(2)	1.484(3)		O3	1.490(2)	1.485(3)
	O1	1.503(2)	1.494(3)		O2	1.497(2)	1.502(3)
**ØB1–O**		**1.49**	**1.48**	**ØB2–O**		**1.49**	**1.48**
Eu–	O6		2.651(2) 4×	B3–	O4	1.463(2)	1.441(3)
	O5		2.665(2) 4×		O1	1.480(2)	1.492(3)
	O1		2.763(2) 4×		O5	1.489(2)	1.464(3)
**ØEu–O**			**2.69**		O3	1.514(2)	1.507(3)
				**ØB3–O**		**1.49**	**1.48**

2.1.5 Bond Angles

Table S6. Interatomic angles /° in $Ga_5B_{12}O_{25}(OH)$ and $In_5B_{12}O_{25}(OH)$ (standard deviations in parentheses).

	**$Ga_5B_{12}O_{25}(OH)$**	**$In_5B_{12}O_{25}(OH)$**		**$Ga_5B_{12}O_{25}(OH)$**	**$In_5B_{12}O_{25}(OH)$**
O4–*M*1–O4	83.57(4)	83.09(8)	O3–*M*2–O3	74.73(5)	69.99(8)
O4–*M*1–O2	85.18(4)	92.63(7)	O7–*M*2–O3	85.04(3)	80.55(4)
O1–*M*1–O6	87.04(4)	84.33(7)	O7–*M*2–O3	85.04(3)	80.55(4)
O4–*M*1–O2	88.14(4)	85.00(7)	O7–*M*2–O3	85.78(3)	84.02(4)
O4–*M*1–O6	88.75(4)	83.44(7)	O7–*M*2–O3	85.78(3)	84.02(4)
O5–*M*1–O6	89.80(4)	85.68(7)	O3–*M*2–O3	88.67(6)	90.8(2)
O4–*M*1–O6	89.82(4)	86.09(7)	O7–*M*2–O3	93.71(3)	94.46(5)
O5–*M*1–O4	90.65(4)	86.81(7)	O7–*M*2–O3	93.71(3)	94.46(5)
O5–*M*1–O2	91.64(4)	93.59(7)	O7–*M*2–O3	94.55(3)	98.75(5)
O4–*M*1–O1	92.51(4)	92.99(7)	O7–*M*2–O3	94.55(3)	98.75(5)
O5–*M*1–O1	93.23(4)	95.36(7)	O3–*M*2–O3	98.31(5)	99.73(9)
O1–*M*1–O2	98.92(4)	107.21(7)	O3–*M*2–O3	98.31(5)	99.73(9)
**ØO–*M*1–O_{90}**	**89.9**	**89.7**	**ØO–*M*2–O_{90}**	**89.8**	**89.7**
O6–*M*1–O2	173.78(4)	168.44(6)	O7–*M*2–O7	168.44(4)	161.15(4)
O5–*M*1–O4	174.22(4)	167.64(7)	O3–*M*2–O3	172.99(3)	168.88(4)
O1–*M*1–O4	174.26(4)	167.38(7)	O3–*M*2–O3	172.99(3)	168.89(4)
**ØO–*M*1–O_{180}**	**174.1**	**167.8**	**ØO–*M*2–O_{180}**	**171.5**	**166.3**
O2–B1–O7	106.6(2)	105.2(2)	O5–B2–O6	107.5(2)	108.6(2)
O6–B1–O1	107.2(2)	108.7(2)	O5–B2–O3	108.8(2)	106.5(2)
O7–B1–O1	108.81(9)	107.2(2)	O6–B2–O3	110.4(2)	112.4(2)
O2–B1–O6	110.8(2)	111.9(2)	O5–B2–O2	108.6(2)	107.4(2)
O2–B1–O1	111.4(2)	109.9(2)	O6–B2–O2	112.0(2)	112.6(2)
O6–B1–O7	112.0(2)	113.7(2)	O3–B2–O2	109.5(2)	109.1(2)
**ØO–B1–O**	**109.5**	**109.4**	**ØO–B2–O**	**109.5**	**109.4**
O1–B3–O5	107.5(2)	107.0(2)			
O4–B3–O1	107.9(2)	107.6(2)			
O4–B3–O5	109.1(2)	109.7(2)			
O1–B3–O3	110.1(2)	109.8(2)			
O5–B3–O3	110.3(2)	111.7(2)			
O4–B3–O3	111.8(2)	110.9(2)			
**ØO–B3–O**	**109.5**	**109.5**			

2.1.6 Hydrogen Bonds

Table S7. Hydrogen bonds /Å,° in $Ga_5B_{12}O_{25}(OH)$ (standard deviations in parentheses).

	D–H	H···A	d(D···A)	D–H–A
O4–H4···O3	0.84(2)	1.8(2)	2.629(2)	156(24)
O4–H4···O6	0.84(2)	2.4(2)	2.786(2)	113(16)
O4–H4···O7	0.84(2)	2.4(2)	2.809(2)	113(17)

2.1.7 Illustration of Displacement Ellipsoids

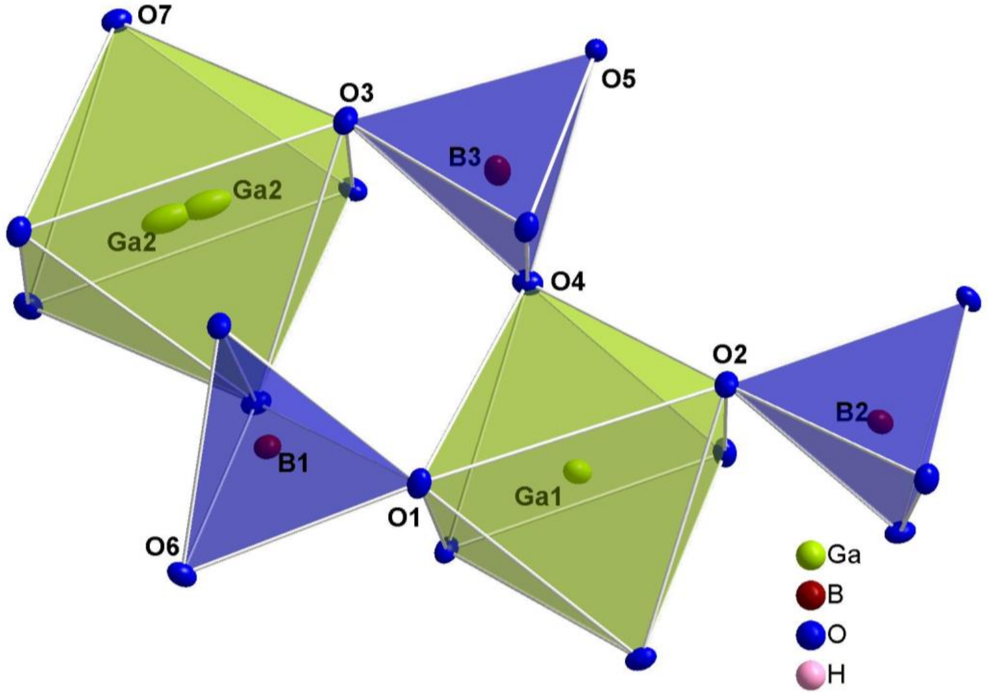

Figure S2. Illustration of the displacement ellipsoids (70 %) of all crystallographically different atoms in $Ga_5B_{12}O_{25}(OH)$ (except the isotropically refined proton).

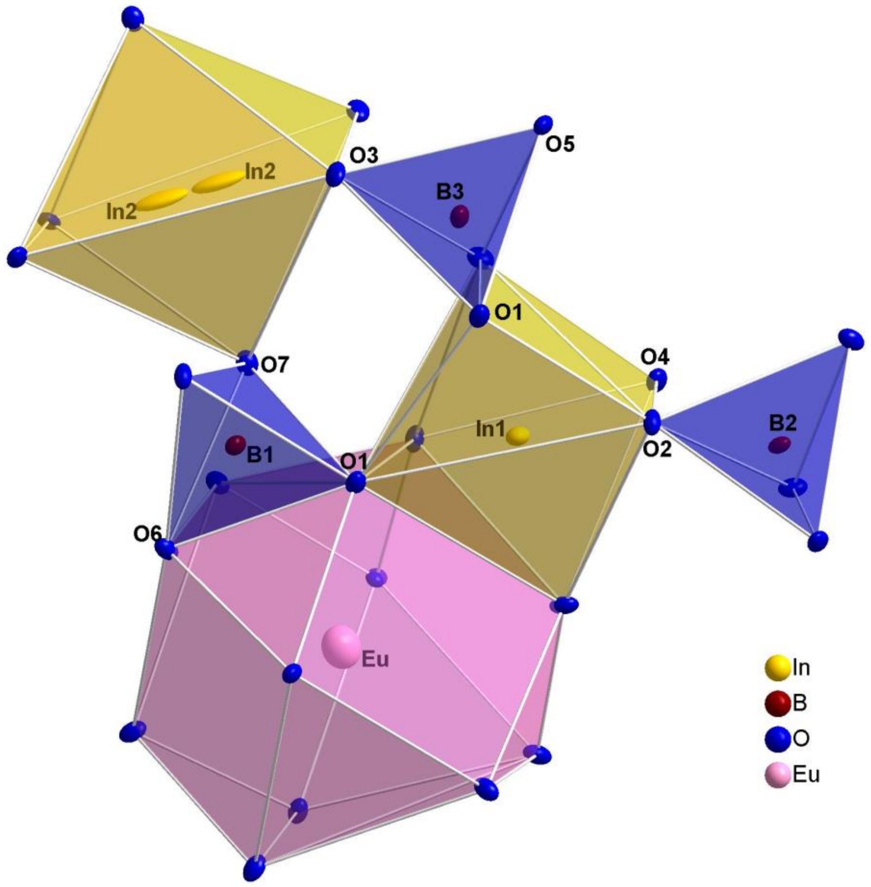

Figure S3. Illustration of the displacement ellipsoids (70 %) of all refined crystallographically different atoms in $In_5B_{12}O_{25}(OH)$.

2.1.8 Structural Comparison with $CaMg_2P_6O_3N_{10}$

Marchuk et al. published an oxonitridophosphate synthesized at high-pressure/high-temperature conditions of 8 GPa and 1100 °C in 2014.[12] $CaMg_2P_6O_3N_{10}$ is built up of a similar condensed tetrahedral network as $M_5B_{12}O_{25}(OH)$ and contains either isolated or edge-sharing double units of octahedra. The edge-sharing octahedra units are filled with Mg and the isolated octahedra with Ca2 atoms that also show oblate like blurred displacement ellipsoids but were located on the symmetric Wyckoff position 8*b* instead of the split refinement in $M_5B_{12}O_{25}(OH)$. The empty or doped cuboctahedral cavities in $M_5B_{12}O_{25}(OH)$ are completely occupied by Ca1 atoms in $CaMg_2P_6O_3N_{10}$. The proton position is not present in the oxonitridophosphate.

2.2 EDX

2.2.1 BSE Image

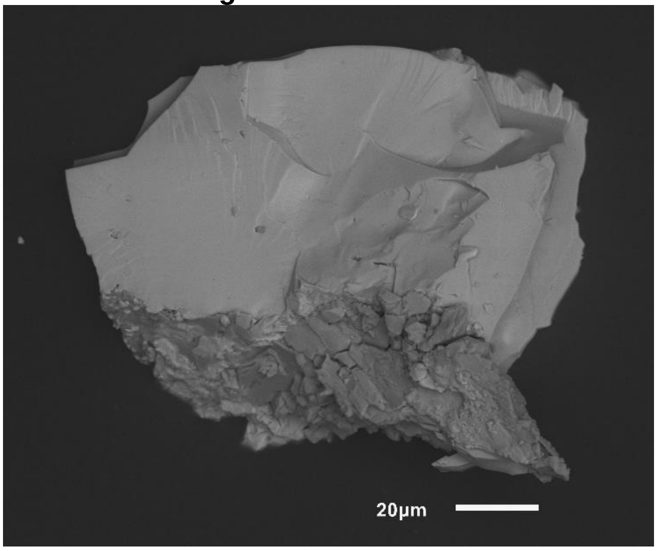

Figure S4. BSE image of the $In_5B_{12}O_{25}(OH):Eu^{3+}$ single-crystal used for the EDX measurement.

2.2.2 EDX Spectrum

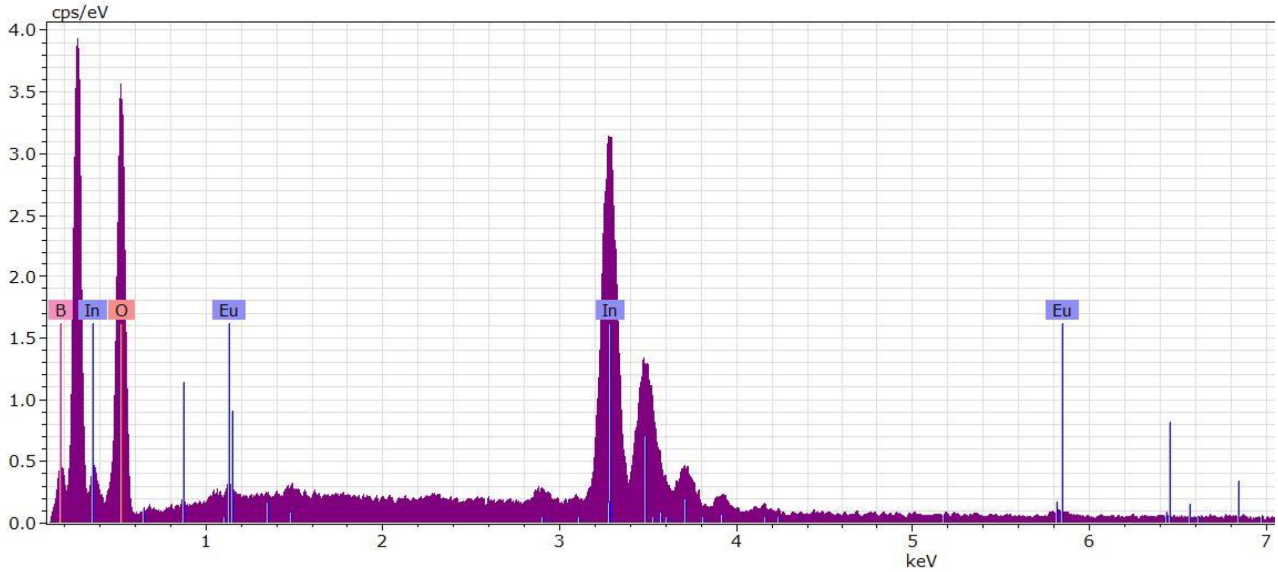

Figure S5. EDX spectrum of $In_5B_{12}O_{25}(OH):Eu^{3+}$. The unindexed high peak at low keV originates from the carbon tape on which the crystal was positioned.

2.2.3 Atom Ratios

Table S8. Measured and expected atom ratios of $In_5B_{12}O_{25}(OH)$:Eu^{3+}. The proton was neglected, the amount of Eu was subtracted from In ($In_{4.98}Eu_{0.02}B_{12}O_{25}(OH)$), and the ratios were normalized to 100 %.

element/ proportion	expected (wt %)	measured (wt %)
In	51.8	44(2)
B	11.7	13(1)
O	36.2	40(1)
Eu	0.3	2(1)

2.3 IR Spectra

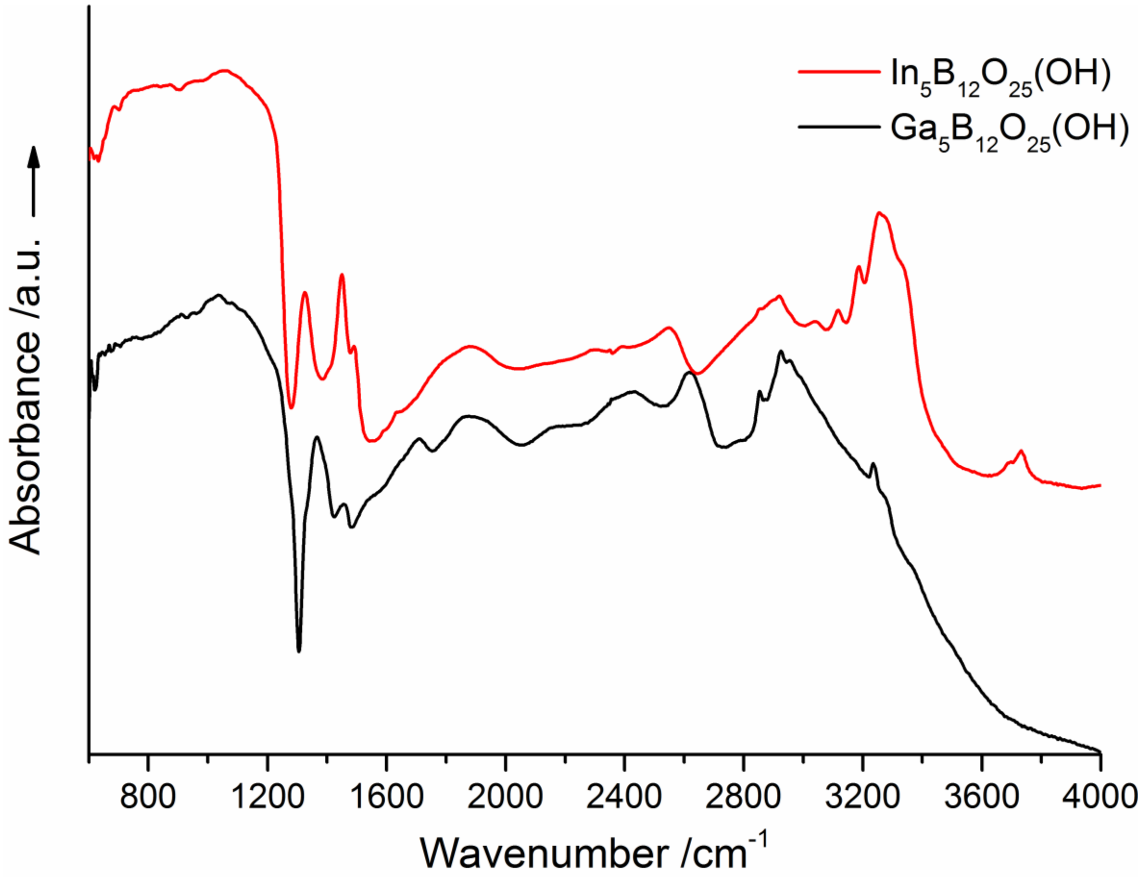

Figure S6. Single-crystal IR spectra of $In_5B_{12}O_{25}(OH)$ and $Ga_5B_{12}O_{25}(OH)$ in the range of 600–4000 cm^{-1}.

2.4 Raman Spectra

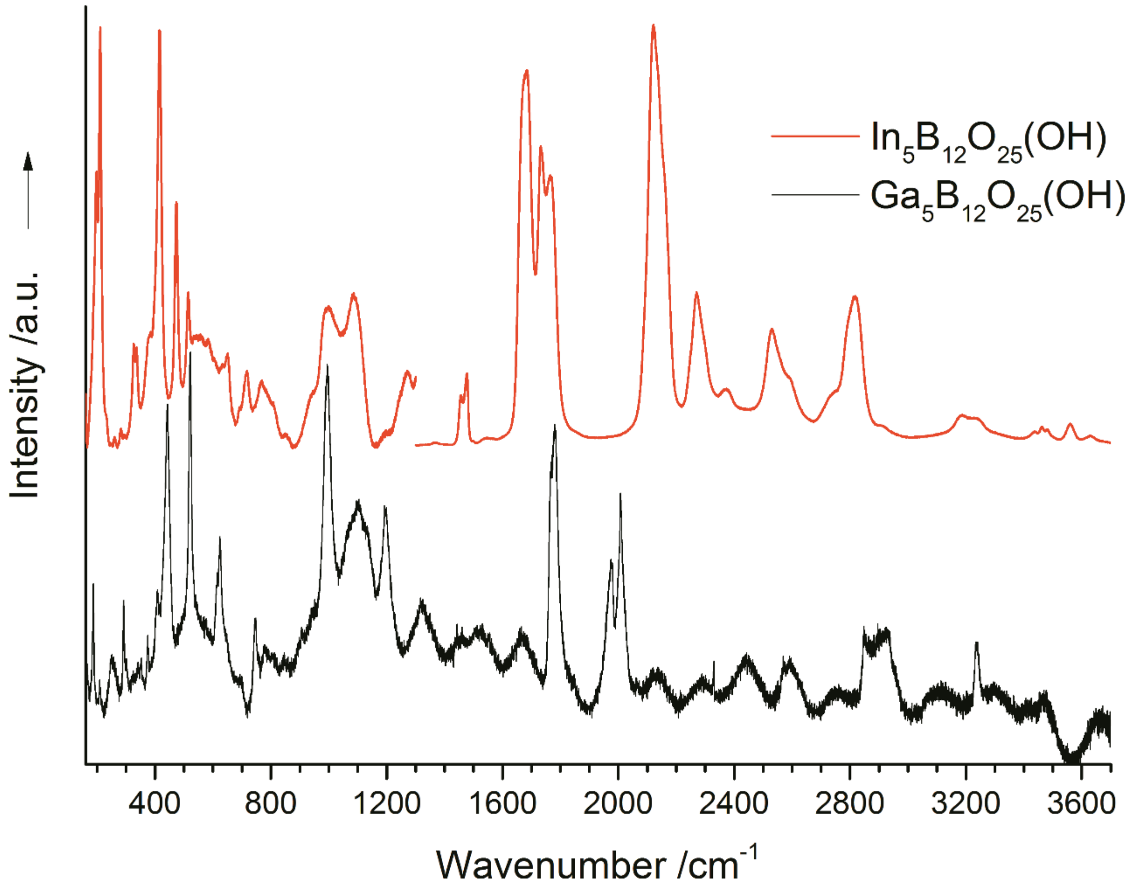

Figure S7. Raman spectra of randomly orientated $In_5B_{12}O_{25}(OH)$ and $Ga_5B_{12}O_{25}(OH)$ single-crystals recorded in the spectral range of 150–3800 cm^{-1}.

3 References

[1] a) H. Huppertz, *Z. Kristallogr. - Cryst. Mater.* **2004**, *219*, 330–338; b) D. Walker, M. A. Carpenter, C. M. Hitch, *Am. Mineral.* **1990**, *75*, 1020–1028; c) D. Walker, *Am. Mineral.* **1991**, *76*, 1092–1100.

[2] D. Vitzthum, S. A. Hering, L. Perfler, H. Huppertz, *Z. Naturforsch.* **2015**, *70b*, 207–214.

[3] D. Vitzthum, L. Bayarjargal, B. Winkler, H. Huppertz, *Inorg. Chem.* **2018**, *57*, 5554–5559.

[4] H. Rietveld, *J. Appl. Crystallogr.* **1969**, *2*, 65–71.

[5] Bruker Analytical X-ray Instruments Inc., Madison, Winsconsin, USA, **2009**.

[6] 2014/5 ed., Bruker AXS Inc., Madison, Wisconsin (USA), **2001**.

[7] a) G. M. Sheldrick, *Acta Crystallogr.* **2008**, *A64*, 112–122; b) G. M. Sheldrick, *Acta Crystallogr.* **2015**, *C71*, 3–8.

[8] L. J. Farrugia, *J. Appl. Crystallogr.* **2012**, *45*, 849–854.

[9] L. Gelato, E. Parthé, *J. Appl. Crystallogr.* **1987**, *20*, 139–143.

[10] A. L. Spek, *Acta Crystallogr.* **2009**, *D65*, 148–155.

[11] Horiba Jobin Yvon S.A.S., Longjumeau Cedex (France), **2010**.

[12] A. Marchuk, L. Neudert, O. Oeckler, W. Schnick, *Eur. J. Inorg. Chem.* **2014**, *2014*, 3427–3434.
